# Mapping Digital Fashion Skills in Green TVET: Foundations for Future Vocational Teacher Competency Framework through Systematic Review and Bibliometric Analysis

**DOI:** 10.12688/f1000research.176893.2

**Published:** 2026-04-22

**Authors:** Roudlotus Sholikhah, Mochamad Bruri Triyono, Sukarno -Sukarno, Sudiyono -Sudiyono, Dias Aziz Pramudita, Hamid Nasrullah

**Affiliations:** 1 Technology and Vocational Education, Graduate School, Universitas Negeri Yogyakarta, Yogyakarta, Special Region of Yogyakarta, 55281, Indonesia; 2 Fashion Design Education, Faculty of Engineering, Universitas Negeri Semarang, Semarang, Central Java, 50229, Indonesia; 3 English Language Education, Faculty of Languages and Arts, Universitas Negeri Yogyakarta, Yogyakarta, Special Region of Yogyakarta, 55281, Indonesia; 4 Mechanical Engineering Education, Faculty of Engineering, Universitas Negeri Semarang, Semarang, Central Java, 50229, Indonesia; 5 Technische Universitat Dresden, Dresden, Saxony, Germany

**Keywords:** Digital fashion skills, Green TVET, vocational teacher competencies, sustainable fashion, systematic literature review, and bibliometric analysis.

## Abstract

**Background:**

The fashion industry is undergoing a digital transformation, creating a pressing demand for digital fashion skills in Green TVET. However, a comprehensive mapping of sustainability-oriented digital fashion skills and their relationship to vocational teacher competency frameworks is still limited. The research conducted in this paper aims to map trends in existing research and identify key digital fashion skills that should be taught in Green TVET, which will be used in designing a digital fashion skills framework for future vocational teachers.

**Methods:**

The method used was a systematic literature review (SLR) using the PRISMA 2020 guidelines. Additionally, bibliometric analysis was used to examine research patterns and the evolution of themes. As a result, 62 peer-reviewed journal articles indexed in Scopus and published between 2021 and 2025 were included according to predetermined inclusion and exclusion criteria.

**Results:**

The core skill domains identified in the digital fashion world for future vocational education teachers in Green TVET resulted in eight skills: 1) Digital Design Skills, 2) Digital Pattern Skills, 3) Digital Production Skills, 4) Digital Sustainability Skills, 5) Digital Ethics & Data Security Skills, 6) Digital Learning & Pedagogy Skills, 7) Digital Communication & Marketing Skills, and 8) Digital Entrepreneurship Skills. Overall, these domains form a competency framework that encompasses the technical, cognitive, and pedagogical dimensions of sustainability-oriented vocational fashion teachers.

**Conclusion:**

This study provides a robust, evidence-based foundation for transforming the fashion vocational curriculum, creating professional development schemes, and strengthening policy measures targeting the development of digital and green skills in vocational education and training (TVET). The proposed framework paves the way for training the next generation of fashion teachers who are ready to face the challenges of digitalization and sustainability.

## Introduction

The rapid digital transformation across various global sectors has significantly changed the skills required in today’s workforce, including the fashion industry. Innovations such as 3D garment simulation, virtual prototyping, artificial intelligence-powered design, and immersive technologies like augmented and virtual reality have transformed the design, production, and education of fashion products. ^1, 2^ Concurrently, the global shift towards sustainability to achieve the Sustainable Development Goals has accelerated the need for environmentally friendly methods in fashion and textile education. ^3– 5^ In this context, Green TVET emerges as a crucial platform for preparing a future workforce equipped with advanced digital skills and a deep understanding of sustainability. Especially for future vocational teacher education, this change emphasizes the importance of developing future vocational educators who master digital mode technologies well into the learning process, while supporting sustainable principles and practices among students. ^2– 4^

Recent research indicates a growing interest in digital skills in vocational and fashion education, reflecting the changing needs of Industry 4.0 and the broader creative economy. Research on digital skills in vocational and technical education emphasizes the importance of technical expertise, innovative thinking, and teaching skills in supporting technology-based learning and preparing students for the workforce. ^6, 7^ In fashion education, research highlights the importance of modern digital technologies such as CLO3D, digital prototyping, and 3D garment simulation in improving design accuracy, minimizing material waste, and accelerating design and production processes. ^8, 9^ Meanwhile, Green TVET emphasizes the importance of building an understanding of sustainability, environmentally friendly skills, and environmentally friendly practices to prepare students for the environmentally friendly sector. ^10, 11^ In line with this, research on vocational teacher training emphasizes the importance of digital teaching skills, flexible skills, and innovative teaching methods to meet the demands of today’s curriculum. ^12, 13^

Although digital technology and sustainability principles have become key concerns in the transformation of the fashion sector and vocational education, there is still a lack of research on the digital fashion skills that prospective vocational teachers must possess to adapt to global change. Previous studies have not provided a comprehensive mapping of digital fashion skills within the context of sustainable vocational education. ^14– 16^ The absence of an evidence-based competency framework makes it difficult for TVET institutions to develop curricula that align with ever-changing industry expectations and technological and sustainability needs. Consequently, many TVET programs struggle to connect technological innovation and digital fashion skills that support sustainability. ^6, 17^ Furthermore, there has been no bibliometric analysis or systematic literature review on global research related to digital fashion skills in the context of sustainable vocational education, especially for vocational education teachers, policy makers, and curriculum designers in making evidence-based decisions. ^18, 19^ To address this issue, a literature review is needed to map digital fashion skills, serving as a foundation for developing a framework and input for the preparation of future vocational teachers within the Green TVET framework.

The integration of digital technologies in fashion education has increasingly been associated with sustainability-oriented competencies, aligning closely with the objectives of Green TVET. Digital fashion tools such as 3D garment simulation, virtual prototyping, and digital patternmaking enable designers and students to visualize and test garment designs without producing multiple physical samples. ^2, 9, 20^ This digital workflow can significantly reduce the consumption of fabric, energy, and other resources typically required in conventional prototyping processes. ^9, 21^ For example, the use of 3D fashion design software such as CLO3D allows garments to be developed, evaluated, and modified virtually before physical production, thereby minimizing material waste and shortening development cycles. Studies have reported that the integration of digital fashion technologies in vocational learning environments can reduce material waste by approximately 35–40% while improving design efficiency and creativity among students. ^9, 21^

From a broader sustainability perspective, the integration of digital technologies in vocational education can also be understood as part of the transition toward greener production systems. The concept of Green TVET emphasizes the development of competencies that enable learners to contribute to environmentally responsible practices, including resource efficiency, sustainable design, and reduced environmental impact across industrial sectors. ^23, 24^ In the context of fashion education, digital design technologies—such as 3D garment simulation, virtual prototyping, and digital patternmaking—offer opportunities to support these objectives by enabling iterative experimentation and product development within virtual environments before physical production takes place. This shift from material-intensive prototyping toward digitally mediated design processes has been widely recognized as a strategy for improving material efficiency and reducing waste in fashion development workflows. ^25, 26^ Therefore, the integration of digital fashion skills within TVET does not merely represent technological advancement in design practice but also contributes to the broader sustainability agenda by equipping future professionals with competencies that support environmentally responsible production systems. Consequently, integrating digital fashion technologies into vocational education may contribute to the development of both digital competencies and sustainability-oriented skills, which are central objectives of Green TVET initiatives aimed at preparing learners for environmentally responsible practices in the fashion industry.

This study uses a Systematic Literature Review and Bibliometric analysis approach to provide an in-depth understanding of how digital fashion skills have evolved, how digital fashion skills relate to sustainability demands, and how this knowledge can assist in designing an appropriate competency framework for vocational teacher education. The novelty of this study also lies in its clear focus on linking technological innovations with sustainability-focused teaching methods, an area that remains under-researched despite its increasing relevance for future-proofing TVET systems. ^6, 11, 27^ By synthesizing existing information into an evidence-based conceptual foundation, this study provides relevant guidance for policymakers, curriculum designers, and teacher educators seeking to update TVET programs in fashion to align with global sustainability and digitalization goals. This view not only emphasizes the importance of empirical research that links theory to practice but also highlights the need for effective strategies in designing teaching models for the future, which can provide a broad range of skills to vocational teachers.

The synthesis resulting from this systematic review and bibliometric analysis provides in-depth insights into the development of digital fashion skills within the broader Green TVET agenda and the impact this direction has on the preparation of future TVET teachers. The research suggests the need for a well-structured competency framework that routinely incorporates digital skills, sustainable design principles, and innovative teaching strategies to keep TVET teachers abreast of industry developments and sustainability demands. ^6, 28, 29^ These findings collectively highlight a strategic direction to provide a robust theoretical and practical foundation for the development of digital-green skills for future TVET teachers.

Research questions of this article are:

RQ1: What are the current trends, themes, and research on digital fashion skills in the context of Green TVET, based on global scientific publications?

RQ2: How are digital fashion skills mapped, specifically in relation to sustainability-oriented fashion in Green TVET?

RQ3: How are competency matrix for digital fashion vocational teachers in Green TVET?

## Methods

### Identification

This research uses the Systematic Literature Review (SLR) method and bibliometric analysis in VOSviewer to analyze the literature on digital skills in vocational education. The PRISMA 2020 guidelines required us to develop a complete search strategy, which we used for transparency and reproducibility purposes. The search terms, combinations, Boolean operators, and database filters used in Scopus are fully reported below ( Table 1). The document shows all search strings that were used to search through the title, abstract, and keywords fields, together with their respective date range, 2021 to 2025, and language restrictions.

**Table 1.** Search strategy.

Database	Field	Search query/Boolean string	Filters	Inclusion criteria	Exclusion criteria
Scopus	Title/Abstract/Keywords	TITLE-ABS-KEY((“digital fashion” OR “fashion digital” OR “virtual fashion” OR “VR/AR fashion”) AND (“skills” OR “competenc*” OR “training”) AND (“TVET” OR “vocational education” OR “technical education”)) AND (LIMIT-TO (PUBYEAR, 2021) OR LIMIT-TO (PUBYEAR, 2022) OR LIMIT-TO (PUBYEAR, 2023) OR LIMIT-TO (PUBYEAR, 2024) OR LIMIT-TO (PUBYEAR, 2025)) AND (LIMIT-TO (LANGUAGE, “English”)) AND (LIMIT-TO (DOCTYPE, “ar”))	Publication Years: 2021–2025; Document Type: Article; Language: English	(1) Peer-reviewed journal articles; (2) Empirical or review studies addressing digital fashion technologies (e.g., VR/AR, digital patternmaking, virtual prototyping); (3) Explicit focus on skills, competencies, training, or curriculum development; (4) Relevant to TVET, vocational, or technical education contexts; (5) Full-text accessible.	(1) Conference papers, book chapters, editorials, notes, or reviews not indexed as articles; (2) Studies unrelated to education or skill development; (3) Conceptual discussions without clear linkage to vocational competencies; (4) Non-English publications; (5) Duplicate records; (6) Studies outside the 2021–2025 publication range.

**Figure 1.**
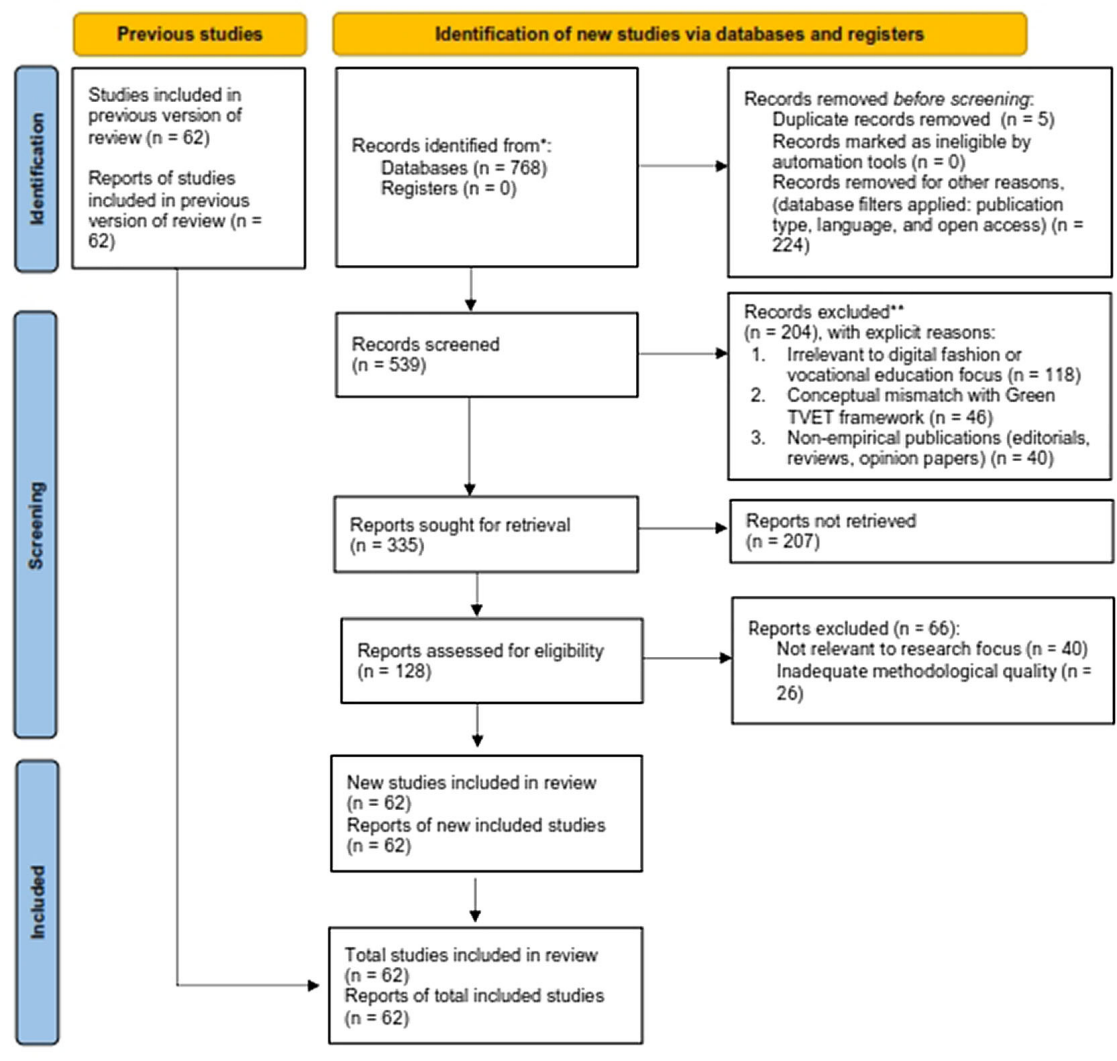
PRISMA flow diagram for the systematic literature review, adapted from the PRISMA 2020 statement. The diagram above illustrates the procedures for identifying, screening, checking for eligibility, and including studies used in this systematic literature review. It also provides a summary of records retrieved from the databases, screening records, the number of articles excluded and their reasons, and the final set of included studies, following the PRISMA 2020 reporting guidelines.

This process is reflected in the following PRISMA flowchart in Figure 1.

The systematic literature review method is demonstrated through the PRISMA Flow Diagram, which is presented in Figure 1. The Scopus database search produced 768 records during its initial search. The screening process began after the team eliminated five duplicate records from the database. The database filtering criteria, which the team established before testing, excluded 224 records because they restricted access to publications between 2021 and 2025 that had to be peer-reviewed, available in English, and accessible through open access. The process of screening titles and abstracts began with 539 records, which remained after the initial screening process. The study objectives determined the screening process, which used specific criteria to include or exclude participants. The analysis of eligible studies revealed three research trends that researchers used to study digital fashion skills development through vocational education and the Green TVET framework.

### Screening

The screening process retained 539 records after all duplicate records and database entries were removed. The researchers conducted title and abstract screening of these records according to their established inclusion and exclusion rules.

The study excluded 204 records because of these specific reasons:

1. 118 records did not relate to digital fashion or vocational education, which was the study’s main focus.
2. 46 records did not match the Green TVET framework and digital skills framework requirements, and
3. 40 records included editorials, opinion papers, and narrative reviews, which were non-empirical publication types.

The researchers requested complete text access for 335 reports after they finished their previous work.

### Eligibility

The researchers requested 335 reports but could only locate 128 documents because 207 reports were lost due to inaccessible full-text content and restricted access. Researchers conducted eligibility testing on 128 available full-text articles.

The full-text assessment phase resulted in 66 article rejections according to documented reasons, which showed three major reasons for rejection. The explicit reasons are documented as follows:

1. lack of direct alignment with the specific research objectives (n = 40), and
2. inadequate methodological quality or insufficient empirical rigor (n = 26).

The research team conducted an extensive eligibility evaluation, which resulted in 62 research studies being selected for inclusion in the systematic review analysis.

### Data abstraction and analysis

The 62 included studies underwent systematic data abstraction using a structured extraction form. The researchers gathered essential data, which included the year of publication, research methodology, study location, research topic, and research methods.

Thematic analysis was used to discover digital skills development patterns that exist in vocational education and the Green TVET framework. This process enabled the synthesis of qualitative insights across studies.

The researchers performed a descriptive quantitative study to analyze how research topics, research methods, and publication patterns changed during the past five years. This complementary analysis provided a broader overview of evolving research priorities and their implications for vocational education policy and practice.

### Coding

The team executed their coding work through a repeated process for all 62 studies that met their eligibility requirements. The team established thematic codes through a process that involved systematic identification of relevant text portions.

The team reviewed their coding decisions throughout the process to establish consistent standards for all coding categories. The team used existing codes to verify their new codes with the analytical framework that they had created to ensure conceptual harmony with their research goals.

The team used an iterative coding method to improve their analytical accuracy while safeguarding their study results from interpretive errors.

## Results

### RQ1: The current trends, themes, and research related to digital fashion skills in the context of Green TVET based on global scientific publications

**Figure 2.**
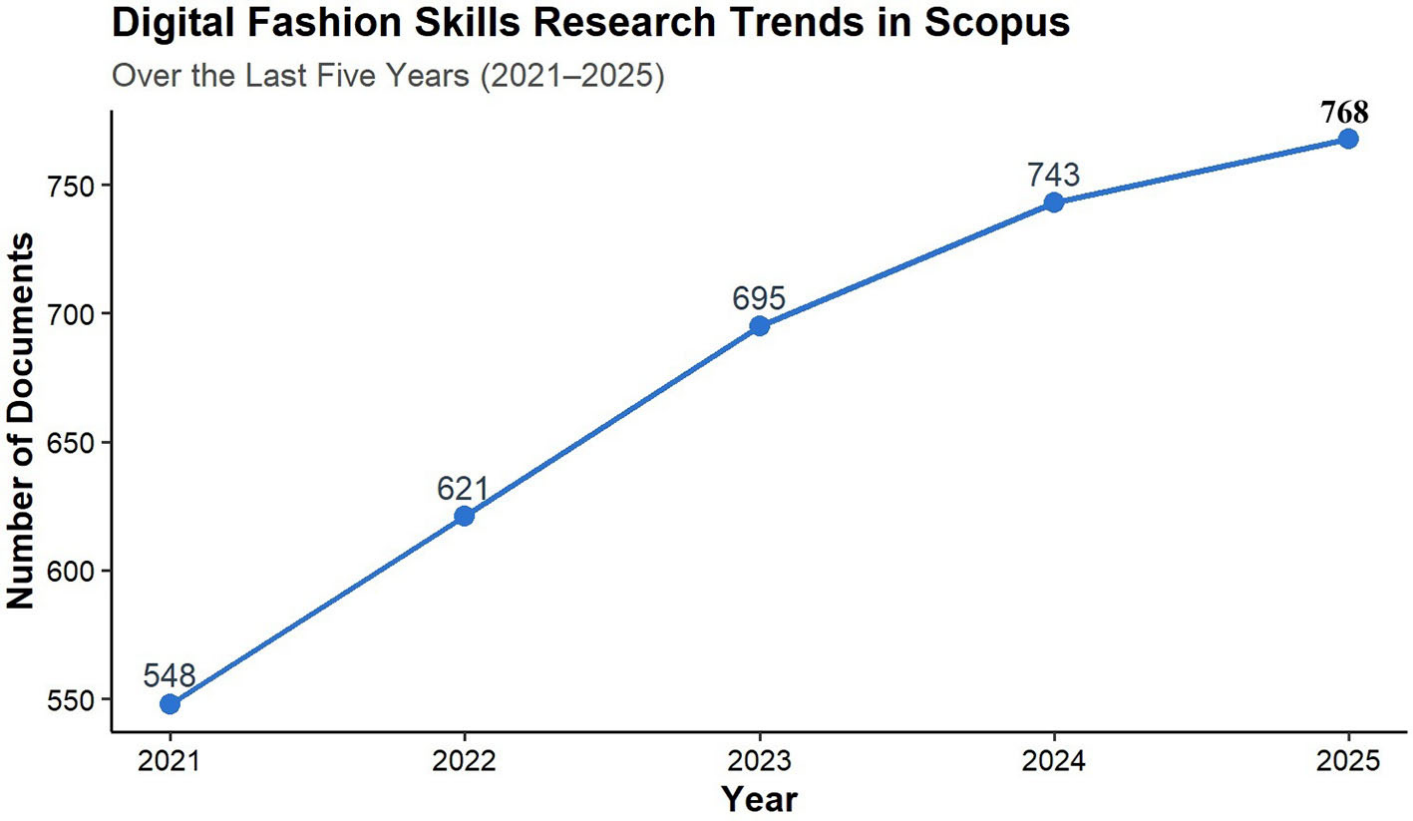
The trend of digital fashion skills publications is shown by the number of documents (articles) published from 2021 to 2025. The figure illustrates the yearly count of published peer-reviewed journal articles from 2021 to 2025, pinpointing the rising curve and escalating academic fascination with digital fashion skills in the setting of fashion education, technology, and sustainability.

According to the research made from the Scopus and Eric databases, the publication frequency in the past five years, from 2021 to 2025, has an increasing trend, as shown in Figure 2. This kind of change reveals different forms of publication output from the years of study in question. A trend in the publication of research associated with digital fashion skills in TVET indicates an annual increase in the number of publications.

**Figure 3.**
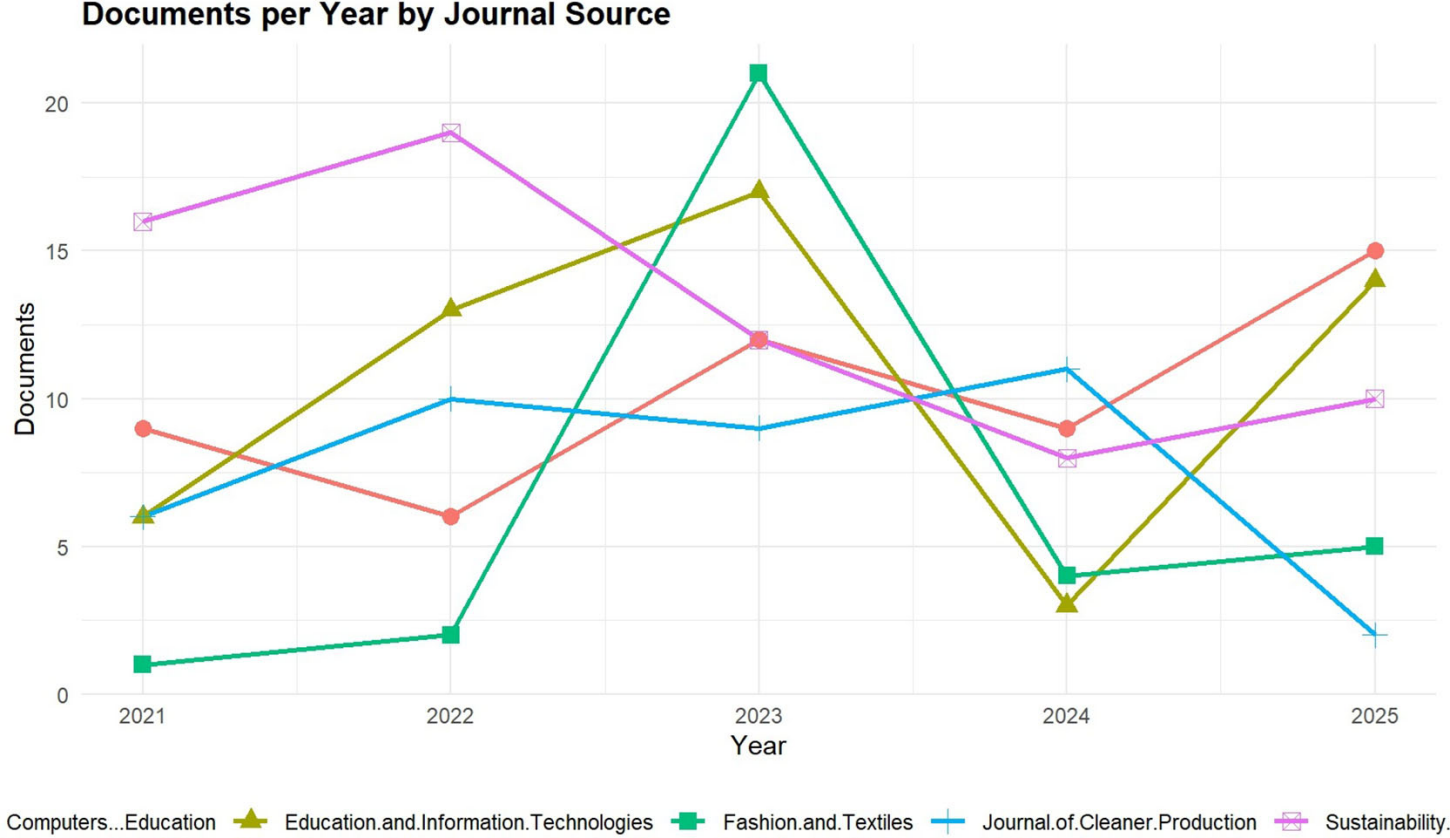
Publication trends by source (2021–2025) in digital fashion skills research. The figure visualizes the spread of writings over various scholarly journals and sources, thus depicting the main channels of digital fashion skills research in the aforementioned time span.

The trends of publication among the five most influential Scopus-indexed sources in Digital Fashion Skills and Green TVET are shown in Figure 3, and the trends are from the year 2021 to 2025. The visualization illustrates a lively scholarly output pattern where sources like Springer Proceedings in Business and Economics and Sustainability Switzerland have kept on releasing high amounts of publications, which is a sign of the increasing academic interest in the areas of digital technologies, sustainability, and education through vocational training. The peaks in the years 2023 and 2025 signal the integration of digital fashion tools like 3D design, virtual prototyping, and AI-driven fashion analytics, etc., into vocational teacher competency frameworks, and thus, teachers’ professional development will be one of the main areas of focus. Besides, the trends also imply that the research works are going to be about the changes in the modes of pedagogical practices towards the use of tech in schools; thus, through the Green TVET ecosystems, the development of educators who are skilled in both sustainability and digital fashion will be possible.

**Figure 4.**
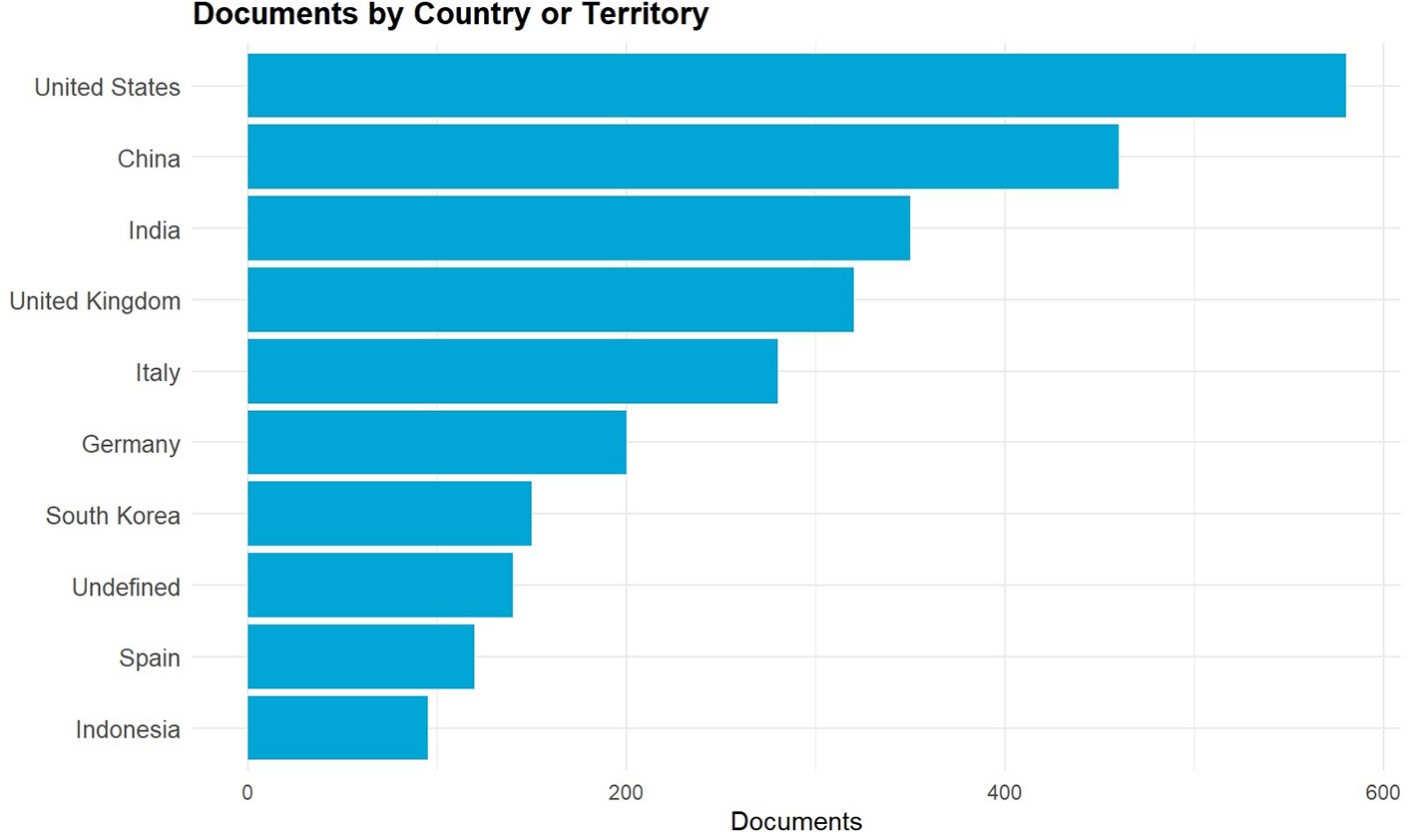
Top 10 contributing countries in digital fashion skills and Green TVET research (2021–2025). The figure shows the research output's geographical distribution by country, thereby indicating the main sources of digital fashion skills and Green TVET-related studies during the past five years.

The graph in Figure 4 shows the distribution of the top ten countries that are the most significant contributors to the worldwide research on Digital Fashion Skills within the discourse of Green TVET from 2021 to 2025. The United States, China, and India rank the highest in terms of contribution and indicate the presence of strong research ecosystems in the areas of digital technology, AI integration, and sustainable innovation. The United Kingdom, Italy, Germany, and Spain are among the European countries that produce significant research outputs, which are an indication of their proactive participation in the digital transformation movement in vocational education, especially by means of virtual fashion design, Industry 4.0 tools, and sustainability-based curricula. South Korea is recognized as a major Asian innovator in the area of digital fashion simulation and virtual garment technologies, among others. The case of Indonesia is particularly interesting as it gradually shows a growing presence that portrays increased academic interest in the field of digital fashion competencies and the development of eco-friendly TVET practices. These countries together lead the world in the areas of digital fashion education, sustainability, and vocational teacher competency development.

**Figure 5.**
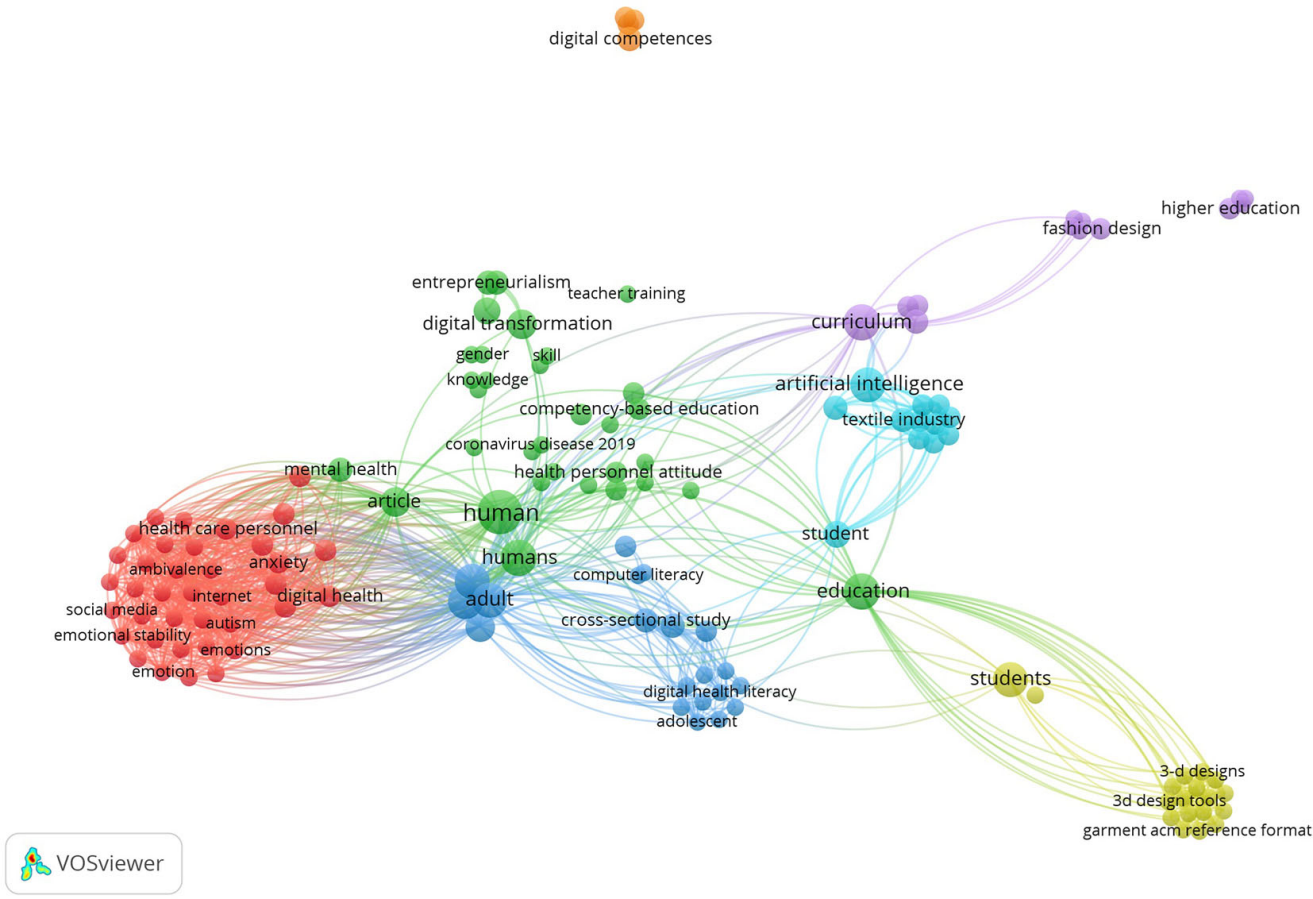
Bibliometric analysis of digital fashion skills keywords using VOSviewer. This figure illustrates a keyword co-occurrence network generated through bibliometric analysis using VOSviewer. Nodes represent the most frequently occurring keywords, while links represent co-occurrence relationships, thus revealing key research topics and digital clusters in the literature related to fashion skills.

A bibliometric mapping derived from co-occurrence keywords produced by VOSviewer is shown in Figure 5. It provides a visual perspective on the research terrain related to digital fashion skills in the Green TVET context.

This bibliometric in Figure 5 reveals the presence of various significant thematic clusters including: (1) a red cluster consisting of the dimensions of mental health, digital literacy, and user behavior; (2) a green cluster consisting of the dimensions of digital transformation, education-based skills, and teacher professional development; (3) a blue cluster consisting of the dimensions of digital literacy, education, and cross-sectional studies; and (4) the yellow and purple clusters consisting of 3D design, the textile sector, fashion design, and higher education. The relationship among the clusters suggests that the development of digital skills in fashion is increasingly associated with AI, curriculum, and students’ digital literacy. In conclusion, these findings attest that research concerning digital fashion skills is evolving in a multidisciplinary way, involving technology, vocational education, and sustainable design innovation that underpins the Green TVET competence framework.

### RQ2: Mapping digital fashion skills, specifically in relation to sustainability-oriented fashion in Green TVET

Mapping 62 studies provides a comprehensive overview of digital fashion skills, which are gradually integrating sustainability aspects into vocational education (Green TVET) as a core characteristic. These studies can be divided into three interrelated competency clusters: technical–production, sustainability–governance, and pedagogy–industry. The technical–production cluster, encompassing Digital Design, Digital Patterning, and Digital Production skills, is the most prominent, perhaps reflecting the field’s focus on 2D/3D fashion CAD, CLO3D simulation, AR/VR-based prototyping, automated digital grading, and AI-assisted garment production. ^9, 30– 32^ These skills are closely related to sustainability in Fashion 4.0, as they result in waste reduction through virtual prototyping, rapid design cycles, and a reduced need for physical samples. ^2^ The same applies to Digital Pattern Skills, specifically zero-waste digital pattern design and virtual fit assessment, among other skills, which have been identified as crucial capabilities that directly integrate environmental friendliness into the design and production process. ^14, 33^

The sustainability and governance cluster has given rise to Digital Sustainability Skills and Digital Ethics & Data Security. These skills are increasingly recognized but remain underrepresented compared to technical skills. Studies continue to integrate zero-waste patterns, eco-friendly prototyping, sustainable material simulation, and digital carbon tracking as necessary skills for fashion model development in TVET. ^34^ Ethical digital practices, copyright literacy, data protection, and secure digital portfolio management are increasingly recognized as essential, given the proliferation of digital assets, AI-based design tools, and online learning platforms. ^35^ However, mapping shows that despite the near-universal acceptance of ethical awareness, there are still few explicit teaching methods for developing these skills in vocational teacher training.

The pedagogy-industry cluster includes Digital Learning & Pedagogy, Digital Communication & Marketing, and Digital Entrepreneurship, which, together with Emerging Technologies and Societies, form the entire spectrum of innovative pedagogy. The most challenging challenge is finding skilled teachers in vocational institutions who are not only able to operate new technologies but also able to create VR-based courses, design digital assessments, and teach CLO3D. ^31, 32^ At the same time, competencies in e-commerce, social media storytelling, and fashion data analytics are indicators of industry demand for graduates who are proficient in digital technologies and also able to operate in omni-channel markets and sustainability-oriented branding. ^2, 8, 36^ Digital Entrepreneurship, although the least discussed aspect, still shows the trend direction in the fashion industry regarding virtual showrooms, AI-based decision-making, and green business models as core components of future environmentally friendly TVET curricula. ^7, 37^

Mapping across all areas reveals a strong but uneven integration of digitalization and sustainability. Technical expertise has been identified as one of the implicit associations that generate positive environmental impacts, for example, through virtual sampling to reduce waste. However, there are very few or no frameworks that address this link, which is reflected in the disparate competencies of teachers in the TVET sector. ^6, 28, 38^ This analysis argues that a robust competency framework for Green TVET educators must integrally combine fluency in advanced digital tools (DDS, DPS, DPrS) with sustainability-oriented digital practices (DSS) and the pedagogical capabilities necessary to create authentic, ethically grounded, and industry-relevant learning experiences (DLPS, DES, DCMS, DEntS). Thus, these results echo international recommendations that vocational teachers should be prepared to combine digital transformation and sustainable development into a future-oriented teaching practice. ^39– 41^

The following is the Digital Fashion Skills Framework, synthesized from a Systematic Literature Review, presented in Table 2.

**Table 2.** Digital fashion skills framework from a systematic literature review.

Authors and Year	Domain skill	Sub-skills identified (Thematic coding)	Code
^19, 42– 48^	**Digital Design Skills**	2D/3D Fashion CAD; AR/VR Simulation; Fabric Rendering; Virtual Garment Visualization	DDS1–DDS4
^1, 49– 53^	**Digital Pattern Skills**	Digital Pattern Drafting Automated Grading; 3D Pattern Simulation Virtual Fit Assessment	DPS1–DPS4
^32, 48, 54– 56^	**Digital Production Skills**	Smart Textile Integration 3D Fashion Printing AI-Based Garment Manufacturing Digital Workflow	DPrS1–DPrS4
^3, 9, 10, 15, 41, 44, 56– 61^	**Digital Sustainability Skills**	Zero-Waste Pattern Making Sustainable Material Simulation; Eco-Friendly Prototyping; Digital Carbon Tracking	DSS1–DSS4
^6, 13, 32, 36, 62^	**Digital Ethics & Data Security Skills**	Copyright Awareness; Data Protection Compliance; Anti-Plagiarism Practice; Secure Digital Portfolio	DES1–DES4
^12, 28, 29, 31, 41, 42, 52, 63– 66^	**Digital Learning & Pedagogy Skills**	E-Learning Content Creation; CLO3D for Teaching; VR-Based Fashion Training; Digital Assessment Design	DLPS1–DLPS4
^68– 74^	**Digital Communication & Marketing Skills**	Social Media Campaigning; E-Commerce Operations; Fashion Data Analytics; Digital Storytelling	DCMS1–DCMS4
^7, 16, 18, 74– 76^	**Digital Entrepreneurship Skills**	Fashion E-Business Models; Virtual Showroom Experience; AI for Fashion Business; Digital Brand Innovation	DEntS1–DEntS4

The thematic analysis results derived from 62 articles indicate that the digital skills map in the sustainability tracker-oriented fashion area, under the context of Green TVET, contains a different yet closely connected competency system. Digital Design Skills was the most important domain, which highlighted the importance of the role of 2D/3D CAD, AR/VR garment simulation, and virtual visualization as the technical capabilities foundation of a highly advanced digital fashion ecosystem in the digital fashion world. Digital Sustainability Skills and Digital Communication & Marketing Skills were the other two highlighted domains portraying the research trend toward zero-waste pattern making, sustainable material simulation, and digital communication literacy, and the analytics of data as the green fashion industry’s main demand. At the same time, domains like Digital Pattern Skills and Digital Learning & Pedagogy Skills brought attention to the requirement for pattern automation and the incorporation of VR, CLO3D, and digital assessment into the methodology of vocational education. Digital Production Skills, Digital Entrepreneurship Skills, and especially Digital Ethics & Data Security Skills were seen to get relatively less attention, and this has revealed that issues of AI-based manufacturing, digital entrepreneurship, and legal and data security are still not fully addressed in research. All in all, these findings validate the unevenness in research priorities while at the same time highlighting the necessity of creating a digital fashion competency framework that is wider, sustainability-driven, and more aligned with the needs of future vocational educators in the Green TVET environment.

**Figure 6.**
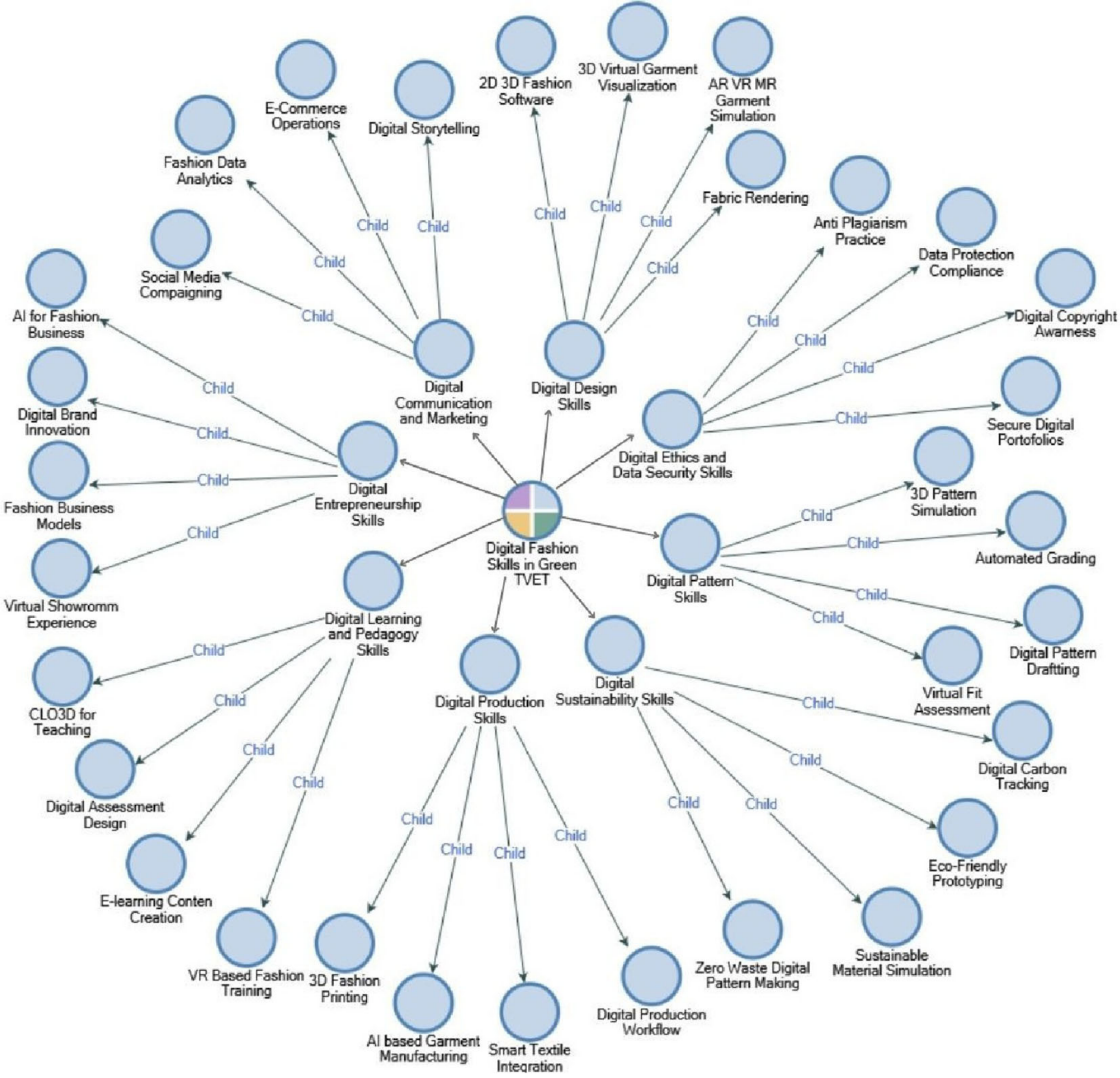
A mapping of digital fashion skills in Green TVET using NVIVO. This figure provides a visualization of the conceptual mapping of essential digital fashion skills in harmony with the Green TVET principles. The technical, digital, and sustainability-oriented competencies are combined in the framework to aid in the development of vocational fashion teacher competency frameworks.

The following is a mapping of digital fashion skills in Green TVET for prospective vocational education teachers based on coding analysis using NVIVO, which is presented in Figure 6.

The mapping of digital fashion skills depicted in Figure 6 portrays an intricate and multidimensional competency ecosystem that perfectly synchronizes with the ongoing revolutionary changes in sustainable and technology-driven fashion education. The framework classifies “Digital Skills in Fashion” as the main competency, surrounded by eight skill clusters that are closely tied to the key domains of Green TVET and future-oriented fashion pedagogy. The Digital Design Skills, Digital Pattern Skills, and Digital Production Skills form the core technological circle of digital fashion competence, including the use of 2D/3D fashion software, CLO3D garment visualization, smart textile integration, automated pattern grading, and AI-supported production workflows. These skills act as a support to the conversion of traditional physical prototyping to totally virtualized, resource-efficient, designer-friendly, and sustainable processes. Apart from the technical skills, the Digital Sustainability Skills also incorporate with eco-responsibility principles via zero-waste digital pattern making, simulating sustainable materials, and tracking digital carbon the very critical elements for instilling environmental consciousness into the fashion workflow.

In addition to technical and sustainability skills, the mapping reveals the professional domains that are crucial for vocational teachers across the board: Digital Learning & Pedagogy Skills and Digital Ethics & Data Security Skills. The demand for teachers to develop e-learning content, conduct VR-based fashion training, protect digital portfolios, and practice ethical digital habits such as copyright protection and data security has been recognized by the winning of these clusters. Besides, the mounting of Digital Communication & Marketing Skills and Digital Entrepreneurship Skills shows that digital business literacy has become the fashion industry’s need. Social media campaigning, e-commerce operations, fashion data analytics, virtual showrooms, and AI-driven innovation are some of the competencies that will prepare future designers and educators for the global digital market. This mapping, therefore, emphasizes that digital fashion skills require software proficiency and support the tech-savvy, sustainable thinking, pedagogy readiness, and entrepreneurship skills fusion that underpins a Green TVET-oriented vocational teacher competency framework’s robust development.

### RQ3: Competency matrix for digital fashion vocational teachers in Green TVET

The combination of mapped digital fashion skills with the thematic categorization of sustainability-oriented competencies led to the establishment of a Competency Matrix that encapsulates the major capability demands for the future vocational fashion teachers in the Green TVET ecosystem. The results point out that the competencies of teachers should not only include technical digital proficiency but should also incorporate a blend of pedagogical, technological, sustainability-driven, and entrepreneurial capabilities. The competency matrix is organized along eight domains—Digital Design, Digital Patternmaking, Digital Production, Digital Sustainability, Digital Ethics & Data Security, Digital Learning & Pedagogy, Digital Communication & Marketing, and Digital Entrepreneurship, each described through the levels of mastery from Beginner to Expert. This organization corresponds to the increasing complexity of skills required for the design of immersive digital fashion learning environments, the integration of AI-based garment technologies, the execution of sustainable virtual workflows, and the assurance of ethical and secure digital practices. Among these are sustainability-oriented competencies like zero-waste digital patterning, material lifecycle simulation, and digital carbon tracking, which are classified as the leading cross-cutting domains necessary for the incorporation of Green TVET principles in teaching practice and the curriculum. The matrix indicates that the expert-level vocational teachers are supposed to perform not only with advanced digital instruments like CLO3D, VR/AR simulation, AI-based production systems, and fashion data analytics, but also to create learning experiences that nurture students’ ecological awareness, digital innovation, and future-oriented industry preparedness. The matrix of competencies gives a systematic, evidence-based framework that can direct the design of curricula, the implementation of professional development programs, and the setting of policy directions aimed at enhancing digital and green skills in fashion vocational education.

The following Competency Matrix for vocational teachers in the fashion field in the context of Green TVET and Digital Fashion is presented in Table 3.

**Table 3.** Competency matrix for vocational teachers in the fashion field in the context of Green TVET and digital fashion.

Competency domain	Core competencies	Specific sub-competencies	Performance indicators
**1. Digital Design Skills**	Ability to design fashion using digital technology	a. 2D/3D Fashion CAD b. AR/VR Simulation for Design c. Fabric Visualization & Rendering d. Virtual Garment Presentation	a. Able to create 2D/3D designs on a CAD platform b. Integrate AR/VR for design exploration c. Display accurate texture visualizations d. Present designs in an interactive digital format
**2. Digital Pattern Skills**	Mastery of precise and efficient digital pattern making	a. Digital Pattern Drafting b. Automated Grading c. 3D Pattern Simulation d. Virtual Fit Assessment	a. Produces production-ready digital patterns b. Automatically grades according to standard sizes c. Simulates patterns onto 3D avatars d. Performs virtual fit analysis
**3. Digital Production Skills**	Capacity to use digital production technology and automation	a. Smart Textile Integration b. 3D Fashion Printing c. AI-Based Manufacturing d. Digital Workflow Management	a. Integrating smart textiles into prototypes b. Operating a 3D printer for fashion c. Using AI in production planning d. Building an end-to-end digital workflow
**4. Digital Sustainability Skills**	Expertise in applying sustainable production principles through digital technology	a. Zero-Waste Patterning b. Sustainable Material Simulation c. Eco-Digital Prototyping d. Carbon Footprint Tracking	a. Digitally design zero-waste patterns b. Simulate eco-friendly materials c. Produce prototypes without physical waste d. Measure carbon impact through software
**5. Digital Ethics & Data Security Skills**	Digital professional ethics and data security in fashion	a. Copyright & IP Literacy b. Data Protection Compliance c. Anti-Plagiarism Practice d. Secure Digital Portfolio	a. Comply with digital design copyright regulations b. Manage student data securely c. Prevent plagiarism in digital works d. Develop a secure digital portfolio
**6. Digital Learning & Pedagogy Skills**	Digital pedagogy for future fashion learning	a. E-Learning Content Development b. CLO3D for Teaching c. VR-Based Fashion Classroom d. Online Assessment Design	a. Creating interactive digital learning content b. Teaching CLO3D effectively c. Managing a VR class for fashion d. Designing a performance-based digital assessment
**7. Digital Communication & Marketing Skills**	Digital communication and marketing for fashion	a. Social Media Campaigning b. E-Commerce Operations c, Fashion Data Analytics d. Digital Storytelling	a. Managing fashion campaigns on digital platforms b. Operating an online store c. Using analytics for design/marketing decisions d. Creating a digital narrative for branding
**8. Digital Entrepreneurship Skills**	Digital entrepreneurship for the future fashion industry	a. Fashion E-Business Models b. Virtual Showroom c. AI for Business Decision d. Digital Brand Innovation	a. Designing a digital fashion business model b. Developing a virtual showroom c. Applying AI to business forecasting d. Generating technology-based brand innovations

The competency matrix from Table 3 created for this research study illustrates the basic areas that the vocational fashion teachers should be skilled to work in a digitally driven and sustainability-oriented Green TVET environment. The domains include many core competencies, particular sub-skills, and performance indicators that can be measured, which are in line with the increasing use of advanced technologies in fashion education. The matrix reveals that teachers are required to be skilled not only in technical areas like digital design, patternmaking, production automation, and sustainability simulation but also in ethical data handling, digital teaching methods, communication, and entrepreneurship. The domains together point out a holistic capability profile in which teachers are anticipated to create interactive digital garments, carry out zero-waste workflows, incorporate AI-enhanced production, and maintain secure digital ecosystems, facilitate VR/AR learning experiences, and mentor students in digital branding and business innovation. Hence, this well-structured framework acts as a comprehensive entrance for upskilling vocational teachers with respect to the industry requirements and sustainability priorities that are global and environmental in the fashion sector.

The eight-domain digital fashion competency framework, which we developed, shows the complete understanding of existing knowledge that connects digital transformation with fashion innovation and Green TVET. The framework exists as a theoretical model that emerged from systematic literature mapping and bibliometric clustering, not from established standards that have received empirical validation. The assessment identifies multiple competency patterns, which include technological proficiency, digital creativity, sustainable design integration, pedagogical adaptability, and industry alignment. These competencies demonstrate how vocational fashion educators will develop new professional capabilities in their future work.

The study establishes a fundamental framework that supports curriculum development and professional training through its organization of various training domains into a competency matrix system. The framework exists as a theoretical model that needs empirical testing to determine its ability to function in different contexts and maintain its essential elements and operational capabilities through various educational environments. Its main value provides a comprehensive framework that researchers can use to develop their theories, policy discussions, and practical evaluations of digital fashion skills within new Green TVET educational frameworks.

## Discussion

The findings of this study contribute to the ongoing digital transformation in Technical and Vocational Education and Training (TVET), particularly in the fashion sector. The findings confirm that trends, digitalization, and sustainability have become foundational in building future-ready vocational competencies. ^2, 48^ Using a systematic review and bibliometric analysis, the authors uncovered that digital fashion technologies, including 3D simulation, AI-assisted design, and immersive VR/AR environments, are being considered by global academics as the most important enablers for making the education ecosystem more efficient, stable, and innovation-driven. However, existing studies remain fragmented and largely limited to a single aspect of technology and competency, without considering a sustainability-oriented competency framework for vocational teachers. This paper contributes to the literature by synthesizing research trends, identifying digital fashion skills, and developing a comprehensive competency matrix based on Green TVET principles.

Bibliometric data analysis reveals a significant and rapid increase in the number of studies on digital fashion technologies, marked by the trend of 3D fashion software and virtual prototyping being incorporated into digital learning worldwide. ^9, 54^ This development aligns with the Fourth Industrial Revolution, ^73^ where digital skills are a necessity in every vocational sector. Global research yields three main clusters: (1) Digital Fashion Design and Visualization, (2) Technology-Enabled Sustainable Fashion Production, and (3) Digital Pedagogy and Learning Innovation in TVET. Papers mentioned in the first cluster draw attention to CLO3D, Browzwear, and AI-enhanced CAD as software that reduces reliance on physical samples and accelerates the design process. ^2^ The second cluster connects the digital world with sustainability arguments, stating that zero-waste patterns, digital prototyping, and smart material simulation are techniques for reducing textile waste and energy consumption. ^9^ The third cluster, on the other hand, emphasizes the importance of digital pedagogy for vocational teachers who need to be trained for new learning environments, particularly those involving VR classrooms, interactive online learning, and online assessment systems. ^43^

These combined trends indicate that digital fashion skills are no longer viewed solely as technical abilities, but rather as a knowledge system encompassing all environmental, pedagogical, and technological aspects. This underscores the need for a more integrated competency framework, such as the one proposed in this study. The analysis revealed that digital fashion skills are closely linked to the sustainability drive characteristic of Green Higher Vocational Education (TVET) and should therefore not be considered solely as technologically literate competencies. According to the authors of a previous study, these are two key aspects of Green TVET: learners are trained in the use of modern technologies, while at the same time, the more environmentally friendly side of the industry is around them, encouraging and leading their development. ^59^ The domains mapped in this study, ranging from digital design and digital patterning to sustainability and digital entrepreneurship, demonstrate the ongoing integration of digitalization and environmental responsibility, a trend that has been recognized but not routinely incorporated into previous studies. Digital Patternmaking and VR-based fitting simulations have led to a decrease in physical sampling needs during prototyping because they create virtual testing methods that designers can use to develop their projects. The extent of waste reduction differs between different situations because the actual amount of waste reduction remains unknown for vocational education settings. ^77, 78^ Meanwhile, digital material simulations allow for the selection of eco-friendly textiles without the need for physical sampling. ^79^ Similarly, AI-powered production planning is considered an approach to data-driven sustainable manufacturing. ^27^ Consequently, the competency mapping output of this study not only confirms but also broadens the scope of previous conceptualizations by developing a structured, interconnected, and mode-specific skills taxonomy for Green TVET educators.

The systematic review has synthesized a competency matrix which reveals the eight competencies that are interrelated mainly and must be acquired by fashion vocational teachers for the Green TVET transformation. The domains of the eight competencies are Digital Design, Digital Patternmaking, Digital Production, Digital Sustainability, Digital Ethics, Digital Pedagogy, Digital Marketing, and Digital Entrepreneurship, which together form a digital industry that is completely aligned with sustainable vocational education. The matrix structure is in tune with the recent literature, which points to the need for multi-faceted competencies among future educators, where digital literacy, ecological consciousness, and pedagogical adaptability are all crucial. ^66^ The matrix is made up of the eight domains, where each domain plays a significant role in the larger aim of teacher preparation, which can incorporate environmentally friendly methods in digital processing. For example, the use of Zero-Waste Patterning, Eco-Digital Prototyping, and Carbon Footprint Tracking directly corresponds to the sustainability capabilities that are part of Green TVET frameworks. ^28^ On the other hand, AI-Based Manufacturing, Virtual Fit Assessment, and VR-Based Fashion Classroom Management are examples of technological advancements that have already taken place in the fashion industry and are reflected in the current fashion industry research. ^2, 81^ This study has significantly improved the previous competency models by establishing sustainability indicators outright within digital competencies. This approach is rare when talking about earlier digital skills taxonomies like DigCompEdu or ISTE standards, and it is this very aspect that makes the matrix the one for vocational fashion contexts only, and at the same time reflective of the reality of the industry’s shift towards green production ecosystems.

The findings of this study also have important implications for policy development within vocational education and training (TVET), particularly in advancing the integration of digital and green competencies in fashion education. As digital fashion technologies such as 3D garment simulation, virtual prototyping, and digital patternmaking become increasingly embedded in industry practices, educational policies should support their systematic integration into TVET curricula as part of sustainability-oriented skill development. Previous studies have emphasized that Green TVET aims to equip learners with competencies that enable environmentally responsible production, resource efficiency, and sustainable innovation across industrial sectors. ^82– 84^ In the context of the fashion industry—one of the most resource-intensive global sectors—the adoption of digital design technologies has been recognized as a promising strategy to reduce physical sampling, optimize material usage, and support more sustainable production workflows. ^85, 86^ Consequently, policy measures may include strengthening digital infrastructure in vocational institutions, supporting teacher professional development in digital fashion technologies, and embedding sustainability-oriented competencies into curriculum standards. Such policy initiatives can help ensure that vocational graduates possess not only advanced digital design capabilities but also the sustainability literacy required to address environmental challenges in contemporary fashion systems.

### Limitations and implications for future research

The review used a systematic and transparent methodological framework, but it contains several limitations that require thorough examination because they affect both knowledge production and field representation. The review depends exclusively on the Scopus database, which provides interdisciplinary indexing and quality control, yet this choice limits the review to a specific citation system used within the database. The reliance on indexing systems for academic visibility creates an advantage for particular geographic areas, publication practices, and knowledge systems, which results in the underrepresentation of local research and emerging academic work that does not meet the standards of mainstream indexing systems. Future reviews would benefit from triangulating multiple databases and repositories to mitigate structural indexing bias and enhance epistemic inclusivity.

The practice of limiting publications to open-access format improves three aspects of research, which include transparency and accessibility, and reproducibility, but it creates a problem because it leads to biased selection of research materials. Different academic fields, funding systems, and worldwide locations show different patterns of open-access publishing. The evidence bases that results from this study shows publishing patterns that limit access to academic work about digital fashion skills and Green TVET, instead of showing all research results. The study shows multiple research problems, which demonstrate how accessibility standards control the main research areas that research studies will show through their systematic reviews.

The period which restricted published works to 2021 until 2025 established modernity for the research findings, yet it restricted the historical development of theoretical frameworks. Vocational education digital transformation exists as a current phenomenon that builds upon past technological advancements and educational practices. The exclusion of earlier foundational works restricts our ability to track how concepts have developed and how theoretical ideas have stayed consistent throughout the discipline.

The researchers used thematic analysis methods, which they applied repeatedly to examine their research results while maintaining both internal consistency and analytical strength. The researchers developed their themes through analytical methods that do not depend on objective methods to collect data. Future research could extend this work through complementary methodological strategies, including bibliometric network analysis, meta-synthesis, or theory-building approaches that critically interrogate how digital and green competencies are conceptualized across educational paradigms. Researchers need to conduct empirical validation studies, which will help them assess digital fashion skill frameworks by evaluating their practical implementation and institutional viability, and their alignment with policies across various economic and international settings. The proposed extensions will enable researchers to develop an understanding of digital transformation, which connects theoretical concepts with actual developments that happen in modern TVET systems worldwide. Future research should validate the proposed framework through empirical testing based on multi-phase expert review, Delphi studies, and pilot implementation studies across various vocational education settings.

## Conclusions

This study provides in-depth insights into the mapping of digital fashion skills within a Green TVET framework through a combination of a systematic review and bibliometric analysis. The results show that global research on digital fashion has shifted dramatically from technology to sustainability, with an increasing focus on 3D simulation, digital prototyping, VR/AR learning environments, and AI-based production systems. This study supports the expansion of technical mastery of digital fashion skills to encompass sustainability principles, ethical data practices, and digital pedagogical skills as necessary for future-oriented vocational education through mapping 62 publications. The eight-domain competency matrix framework not only provides a structural and comprehensive map but also positions vocational teachers in a core position to operationalize the processes of ecological awareness, digital innovation, and pedagogical change in the fashion education sector. In other words, this study is a step forward in current research by defining integrated competencies design, production, pedagogy, sustainability, entrepreneurship, and ethics, which are largely lacking in the existing TVET literature. Thus, this paper sets a reference point for reorienting vocational teacher training towards a digitally empowered and sustainability-aligned future.

The findings of this study provide the basis for several strategic recommendations to policymakers, technical and vocational education and training (TVET) institutions, and future researchers. First and foremost, the proposed competency matrix should be integrated into the curriculum development of TVET institutions so that digital sustainability skills, namely, zero-waste patterns, eco-digital prototyping, and carbon tracking, are clearly stated in learning outcomes and learning activities. This way, digital sustainability skills will be integrated with learning outcomes and teaching methods. Second, teacher education programs require systematic professional development and support, which not only provides vocational teachers with expertise in 3D modeling software, immersive VR classrooms, AI-assisted manufacturing, and digital entrepreneurship but also ensures that these teachers stay abreast of industry standards and sustainability demands. Third, the government should accelerate the digital-green transition in TVET by providing the necessary supporting infrastructure, industry partnerships, and a regulatory framework that will not only facilitate the use of digital modeling technologies but also ensure their sustainable use.

Future research should confirm the competency matrix with empirical evidence using expert judgment, pilot implementation, or mixed-method teacher readiness assessments. Furthermore, cross-country comparisons are recommended to analyze the integration of digital sustainability competencies across national TVET systems. Furthermore, researchers should consider developing digital assessment tools and microcredentialing systems to support competency-based lifelong learning for TVET teachers in the green digital era. With these directions in mind, future research can refine and expand the framework to ensure its relevance and practical applicability in changing educational and industrial contexts.

## Use of AI tools

During the preparation of this manuscript, Grammarly was used for language editing and grammatical refinement only. The use of this tool did not influence the study design, data analysis, interpretation of results, or the scientific content of the article. The authors take full responsibility for the content of the manuscript.

## Data Availability

All data underlying the results are available as part of the article, and no additional source data are required. Extended data supporting this study are available in the Figshare repository https://doi.org/10.6084/m9.figshare.31021585. ^87^ The dataset includes the systematic literature review data extraction table, bibliographic records, screening outcomes, and supporting materials used during the eligibility assessment process. Data are available under the terms of the Creative Commons Attribution 4.0 International license (CC-BY 4.0). This study adheres to the PRISMA 2020 (Preferred Reporting Items for Systematic Reviews and Meta-Analyses) guidelines. The completed PRISMA 2020 checklist and PRISMA flow diagram are available via Figshare. Figshare: Supporting Data for a Systematic Literature Review on Digital Fashion Skills Mapping in Green TVET. https://doi.org/10.6084/m9.figshare.31021585 ^87^ Data are available under the terms of the Creative Commons Attribution 4.0 International license (CC-BY 4.0).
