# Supplementary material for: Mapping Digital Fashion Skills in Green TVET: Foundations for Future Vocational Teacher Competency Framework through Systematic Review and Bibliometric Analysis
Source: F1000Res. 2026 Apr 22;15:196. Originally published 2026 Feb 5. [Version 2] doi: 10.12688/f1000research.176893.2 (PMC13146484; doi:10.12688/f1000research.176893.2)
Supplement: Peer review history

# Reviewer report: Reviewer response for version 2

**DOI:** 10.5256/f1000research.197555.r477620

**Published:** 2026-05-05

**Authors:** Winwin Wiana

**Affiliations:** 1 Universitas Pendidikan Indonesia, Bandung, West Java, Indonesia

After reviewing the revised manuscript, I find that the discussion is sufficiently comprehensive and aligned with the issues addressed.

 I recommend that the manuscript be accepted for indexing.

Are the rationale for, and objectives of, the Systematic Review clearly stated?

Yes

Is the statistical analysis and its interpretation appropriate?

Yes

If this is a Living Systematic Review, is the ‘living’ method appropriate and is the search schedule clearly defined and justified? (‘Living Systematic Review’ or a variation of this term should be included in the title.)

Yes

Are sufficient details of the methods and analysis provided to allow replication by others?

Yes

Are the conclusions drawn adequately supported by the results presented in the review?

Partly

Reviewer Expertise:

fashion design

I confirm that I have read this submission and believe that I have an appropriate level of expertise to confirm that it is of an acceptable scientific standard.

# Reviewer report: Reviewer response for version 2

**DOI:** 10.5256/f1000research.197555.r477619

**Published:** 2026-04-27

**Authors:** Indarti Indarti

**Affiliations:** 1 Universitas Negeri Surabaya, Surabaya, East Java, Indonesia

I agree that this article should be indexed and i approve of this manuscript.

Are the rationale for, and objectives of, the Systematic Review clearly stated?

Partly

Is the statistical analysis and its interpretation appropriate?

Yes

If this is a Living Systematic Review, is the ‘living’ method appropriate and is the search schedule clearly defined and justified? (‘Living Systematic Review’ or a variation of this term should be included in the title.)

Yes

Are sufficient details of the methods and analysis provided to allow replication by others?

Yes

Are the conclusions drawn adequately supported by the results presented in the review?

Yes

Reviewer Expertise:

NA

I confirm that I have read this submission and believe that I have an appropriate level of expertise to confirm that it is of an acceptable scientific standard.

# Reviewer report: Reviewer response for version 1

**DOI:** 10.5256/f1000research.195015.r456338

**Published:** 2026-03-05

**Authors:** Winwin Wiana

**Affiliations:** 1 Universitas Pendidikan Indonesia, Bandung, West Java, Indonesia

**Introduction**

 In the Introduction section, the author attempts to present the phenomenon of Green TVET as an integral part of digital skills in vocational and fashion education. However, there is one concept that seems to have low relevance between the mastery of digital skills in the field of fashion and Green TVET's emphasis on environmentally friendly skills. Is there a significant relevance between digital technology learning activities in fashion (such as CLO3D or other digital tools) and their impact on environmentally friendly skills? Please explain and provide some empirical examples.

 In conclusion, the author states that “..........strengthening policy measures targeting the development of digital and green skills in vocational education and training (TVET).  This condition is not elaborated well in the discussion section, so the above statement needs to be discussed in more detail.

Are the rationale for, and objectives of, the Systematic Review clearly stated?

Yes

Is the statistical analysis and its interpretation appropriate?

Yes

If this is a Living Systematic Review, is the ‘living’ method appropriate and is the search schedule clearly defined and justified? (‘Living Systematic Review’ or a variation of this term should be included in the title.)

Yes

Are sufficient details of the methods and analysis provided to allow replication by others?

Yes

Are the conclusions drawn adequately supported by the results presented in the review?

Partly

Reviewer Expertise:

fashion design

I confirm that I have read this submission and believe that I have an appropriate level of expertise to confirm that it is of an acceptable scientific standard, however I have significant reservations, as outlined above.

# Response

**Published:** 2026-03-09

**Authors:** Roudlotus Sholikhah

**Affiliations:** Graduate School, Universitas Negeri Yogyakarta, Yogyakarta, Special Region of Yogyakarta, Indonesia

We sincerely thank the reviewer for the constructive and insightful comments, which have helped improve the conceptual clarity and policy relevance of the manuscript. The manuscript has been revised accordingly as outlined below.

 1. Relevance between Digital Fashion Skills and Green TVET

 Thank you for highlighting the need to clarify the relationship between digital fashion technologies and Green TVET. In response, we have strengthened the Introduction section by providing a clearer theoretical explanation of how digital fashion technologies—such as 3D garment simulation, virtual prototyping, and digital patternmaking—can contribute to sustainability-oriented competencies in vocational education. The revised text now explains that digitally mediated design processes can reduce reliance on physical sampling, improve material efficiency, and support environmentally responsible production practices in fashion development. In addition, several relevant studies have been incorporated to provide empirical examples supporting the linkage between digital fashion technologies and sustainability practices.

 2. Elaboration of Policy Measures for Digital and Green Skills in TVET

 We appreciate the reviewer’s suggestion to further elaborate on the policy implications mentioned in the Conclusion. To address this comment, an additional paragraph has been added to the Discussion section to clarify how the findings of this study relate to policy development in TVET systems. The revised discussion now outlines potential policy measures, including strengthening digital infrastructure in vocational institutions, enhancing teacher professional development in digital fashion technologies, and integrating sustainability-oriented competencies into TVET curricula. These revisions ensure that the statement in the Conclusion is now supported by a clearer and more detailed discussion.

 We are grateful for the reviewer’s valuable feedback, which has significantly strengthened the conceptual coherence and practical implications of this study.

# Reviewer report: Reviewer response for version 1

**DOI:** 10.5256/f1000research.195015.r456337

**Published:** 2026-02-27

**Authors:** Indarti Indarti

**Affiliations:** 1 Universitas Negeri Surabaya, Surabaya, East Java, Indonesia

The topic is timely and relevant, particularly given the rapid integration of digital technologies (e.g., 3D design, AI, VR/AR) in fashion education and the growing emphasis on sustainability in vocational systems. The attempt to synthesize literature trends and translate them into a structured competency framework is commendable and potentially impactful.

 However, several methodological and reporting issues require substantial clarification before the manuscript can be considered robust and reproducible.

1. Clarity and Consistency of Data Sources. The manuscript references Scopus as the primary database for the 62 selected studies, but other sections mention ERIC in relation to trend analysis.
2. Search Strategy Transparency and Reproducibility. For a systematic review aligned with PRISMA 2020 standards, the complete search strategy must be reported, preferably in a table or appendix.
3. PRISMA Flow and Screening Logic. Providing explicit exclusion reasons (e.g., irrelevance, conceptual mismatch, non-empirical design) would significantly strengthen methodological rigor.
4. Open-Access Restriction and Potential Bias. Discuss its implications in the limitations section.
5. Validation of the Proposed Competency Framework. The eight-domain framework and competency matrix are derived from literature synthesis, but no external validation (e.g., expert consultation, Delphi method, stakeholder review) has been conducted. At minimum, the framework should be clearly positioned as a *proposed conceptual model* pending empirical validation.
6. Strength of Quantitative Claims. Some claims regarding sustainability impact (e.g., waste reduction percentages through VR-based prototyping) should be carefully contextualized and directly supported by clearly cited empirical evidence.
7. Terminology should be standardized (e.g., “Digital Pattern,” “Digital Patterning,” “Digital Patternmaking”).

Are the rationale for, and objectives of, the Systematic Review clearly stated?

Partly

Is the statistical analysis and its interpretation appropriate?

Yes

If this is a Living Systematic Review, is the ‘living’ method appropriate and is the search schedule clearly defined and justified? (‘Living Systematic Review’ or a variation of this term should be included in the title.)

Yes

Are sufficient details of the methods and analysis provided to allow replication by others?

Yes

Are the conclusions drawn adequately supported by the results presented in the review?

Yes

Reviewer Expertise:

fashion design; creative industry

I confirm that I have read this submission and believe that I have an appropriate level of expertise to confirm that it is of an acceptable scientific standard, however I have significant reservations, as outlined above.

# Response

**Published:** 2026-03-09

**Authors:** Roudlotus Sholikhah

**Affiliations:** Graduate School, Universitas Negeri Yogyakarta, Yogyakarta, Special Region of Yogyakarta, Indonesia

We sincerely thank the reviewers for their constructive and insightful feedback. The manuscript has been carefully revised to address all comments. The revisions have strengthened the methodological clarity, conceptual position, and internal consistency of the studies. Detailed responses are provided below.

 1.    Clarity and Consistency of Data Sources

 We have clarified in the Methods section (Data Sources and Selection Procedures) that Scopus was the sole database used for the final selection of 62 studies. Ambiguous references to ERIC have been removed to ensure full consistency and transparency in the data collection process.

 2.    Transparency of Search Strategy (PRISMA 2020 Compliance)

 The complete search strategy has now been added to Table 1, Search Strategy, including Boolean operators, search strings, keywords, date ranges, language restrictions, and inclusion/exclusion criteria. The screening steps have also been clarified in the Methods section to enhance transparency and reproducibility in accordance with PRISMA 2020 standards.

 3.    PRISMA Flowchart and Exclusion Logic

 The PRISMA 2020 flowchart (Figure 1) has been revised to include explicit exclusion reasons (e.g., irrelevance, conceptual-only studies, duplicates, non-English language publications). The screening logic and numerical consistency between the diagram and textual descriptions have been clarified in the Methods section.

 4.    Open Access Restrictions and Potential Bias

 A specific discussion has been included in the “Limitations and Implications for Future Research” section, which addresses potential publication and indexing bias arising from open access restrictions and reliance on a single database, along with their implications for representativeness and generalizability.

 5.    Validation of the Proposed Competency Framework

 The eight-domain competency framework has been explicitly repositioned as a conceptual model derived from a systematic literature synthesis in the Discussion (Framework Integration) section. We now clearly state that no external validation (e.g., Delphi method, expert consultation, field testing) was conducted within the scope of this study and that empirical validation is needed in future research.

 6.    Strength of Quantitative Claims

 All quantitative statements related to sustainability impacts have been carefully reviewed and revised in the Discussion (Sustainability Implications) section. Overly general numerical claims have been contextualized or reframed to reflect context-dependent findings to avoid overstatement.

 7.    Standardization of Terminology

 Terminology has been standardized throughout the manuscript. The term “Digital Patternmaking” is now used consistently throughout the text, tables, figures, and competency matrix to avoid conceptual ambiguity.
